# Characterization of Species- and Sex-Dependent Volatile and Non-Volatile Flavor Profiles in Three Commercially Important Mussel Species

**DOI:** 10.3390/foods15162915

**Published:** 2026-08-20

**Authors:** Ruoshu Li, Xiaojian Xiu, Jiangbo Xiang, Wenhui Qu, Lipin Chen, Xinyue Wang, Xiaoming Jiang, Hongying Liu, Tingyu Feng, Changhu Xue

**Affiliations:** 1 College of Food Science & Engineering, Ocean University of China, Qingdao 266404, China; 2 Qingdao Institute of Marine Bioresources for Nutrition & Health Innovation, Qingdao 266109, China; 3 Quanzhou Institute of Marine Bioresources Industry, Quanzhou 362700, China; 4 China Resources Wind Power (Shanwei) Co., Ltd., Shanwei 516600, China; 5 College of Food Science and Technology, Hainan University, Haikou 570228, China

**Keywords:** cultivated mussel, species differentiation, sex differences, free amino acids, GC-IMS, flavor compounds

## Abstract

The flavor characteristics of three commercially important, cultivated mussel species (*Mytilus coruscus*, *Mytilus galloprovincialis*, and *Perna viridis*) were investigated through analyses of free amino acids, organic acids, 5′-nucleotides, fatty acids, and volatile organic compounds (VOCs). Distinct differences among species–sex groups were observed in both volatile and non-volatile flavor compounds. *M. coruscus* and *M. galloprovincialis* showed greater umami potential than *P. viridis*. Female *M. galloprovincialis* exhibited the highest equivalent umami concentration (EUC) (2.56 ± 0.21 g MSG/100 g), whereas male *M. galloprovincialis* and male *M. coruscus* contained the highest levels of sweet-tasting amino acids (605.42 ± 28.72 mg/100 g) and umami-tasting amino acids (209.29 ± 13.31 mg/100 g), respectively. Gas chromatography–ion mobility spectrometry (GC-IMS) analysis identified 67 volatile compounds, and 12 key discriminative volatiles were selected, including three aldehydes, four ketones, three alcohols, 2-ethylfuran, and acetic acid. Males generally showed higher omega-3 polyunsaturated fatty acids (ω-3 PUFAs)and omega-6 polyunsaturated fatty acids (ω-6 PUFAs) proportions than females, with the highest ω-3 PUFAs proportion in male *M. coruscus* (45.12 ± 0.22%) and the highest ω-6 PUFAs proportion in male *P. viridis* (8.13 ± 0.30%). Correlation analysis suggested that ω-6 PUFAs were more closely associated with aldehydes, whereas ω-3 PUFAs were more closely associated with alcohols and ketones. These associations provide preliminary evidence for understanding flavor differences among cultivated mussel groups.

## 1. Introduction

Mussels, belonging to the family *Mytilidae*, are among the most economically important marine bivalves cultivated worldwide. In China, *Mytilus coruscus*, *Mytilus galloprovincialis*, and *Perna viridis* are the major cultured mussel species and contribute substantially to shellfish production [1]. Highly valued for their nutrition and flavor, mussels are widely consumed and increasingly used in functional foods [2,3]. Flavor determines consumer preference and market competitiveness [4]. Seafood flavor can be influenced by various factors, such as diet, habitat, season, geographic origin, reproductive stage, and sex [5,6,7]. Della et al. showed that cultivation site influenced the nutritional composition of *M. galloprovincialis* [8]. *M. coruscus* is widely distributed and cultivated along both northern and southern coastal regions; *M. galloprovincialis* predominates in northern waters, whereas *P. viridis* is mainly farmed in warm southern coastal regions [9,10].

During gonadal development, sex-related differences in reproductive investment, energy allocation, and gonadal composition may alter flavor-related compounds, such as amino acids, nucleotides, fatty acids, and volatile compounds, resulting in distinct male and female flavor profiles [4,5,11]. Zhang et al. reported significant sex-related differences in the lipid and fatty acid profiles of *M. edulis*, with males showing a higher combined proportion of eicosapentaenoic acid (EPA) and docosahexaenoic acid (DHA) than females [12]. Zhao et al. reported that sex affected volatile compound and metabolite formation during thermal processing [4]. In addition, species and sex are often investigated separately, leaving their combined effects on volatile and non-volatile flavor compounds largely overlooked [13]. The relationships among fatty acids, volatile compounds, and non-volatile taste-active substances, which are closely associated with flavor formation, also remain unclear. Therefore, comparative flavor analysis across species and sexes is necessary to better understand flavor variation in cultivated mussels.

Flavor is an essential quality of shellfish, determining their acceptability and consumer preference [14]. Flavor perception results from the integration of aroma and taste [15]. Aroma is mainly associated with volatile organic compounds (VOCs), whereas taste is primarily determined by non-volatile compounds, including free amino acids (FAAs), organic acids, and 5′-nucleotides [16,17,18,19]. Flavor-related compounds are generated or accumulated through lipid oxidation, enzymatic reactions, protein hydrolysis, microbial metabolism, and Maillard reactions, while changes in physicochemical properties may further influence their formation and transformation [20]. In seafood, FAAs, 5′-nucleotides, and organic acids contribute particularly to umami and sweet tastes [18,21]. Therefore, the combined evaluation of volatile compounds, non-volatile taste-active substances, and fatty acid composition provides a more comprehensive characterization of seafood flavor.

Compared with Gas chromatography–mass spectrometry (GC-MS), Gas chromatography–ion mobility spectrometry (GC-IMS) offers rapid analysis, high sensitivity, intuitive visualization, and relatively simple sample preparation, making it suitable for profiling VOCs in aquatic products [20]. GC-IMS combined with chemometrics has also been used to monitor VOC changes in live *P. yessoensis* under different packaging conditions, demonstrating its potential to relate physiological vitality to flavor quality and support quality control throughout the live seafood supply chain [22]. Zhang et al. reported that GC-IMS could discriminate samples subjected to different storage temperatures and storage times [23]. Zhao et al. used GC-IMS to distinguish volatile profiles between male and female mussels and identified aldehydes, ketones, and alcohols as characteristic volatile compounds [4].

This study aimed to comprehensively evaluate the flavor characteristics of three commercially important mussel species (*M. coruscus*, *M. galloprovincialis*, and *P. viridis*) by considering both species- and sex-related differences. Free amino acids, organic acids, 5′-nucleotides, fatty acids, and VOCs were systematically analyzed to characterize taste and aroma attributes. Correlation analyses elucidated the potential links among fatty acids, volatile compounds, and non-volatile flavor substances. The results provide insights into flavor variation across mussel species and sexes.

## 2. Materials and Methods

### 2.1. Sample Collection and Preparation

Live *M. coruscus* were collected from Rizhao, Shandong Province; *M. galloprovincialis* were collected from Qingdao, Shandong Province; and *P. viridis* were collected from Quanzhou, Fujian Province. All live cultivated mussels, approximately 2–3 years old, were collected between February and March 2025. This sampling period generally corresponds to active gonadal development before the main spring spawning season [9,10,24,25]. For all three species, sex was determined macroscopically based on gonadal coloration: orange-yellow gonads were classified as female and milky-white gonads as male [12]. Representative gonadal tissues of *M. coruscus* were further examined by hematoxylin and eosin staining to support sex identification and characterize gonadal development (Figure S1). The mean shell length, shell width, shell height, and body mass were 88.69 ± 5.14 mm, 44.56 ± 1.12 mm, 29.90 ± 1.49 mm, and 49.78 ± 6.46 g for *M. coruscus*; 60.92 ± 2.35 mm, 30.52 ± 1.74 mm, 22.70 ± 1.20 mm, and 17.91 ± 2.97 g for *M. galloprovincialis*; and 83.11 ± 3.58 mm, 35.09 ± 1.70 mm, 21.98 ± 1.81 mm, and 28.00 ± 3.88 g for *P. viridis*, respectively.

For each sampling, intact and live mussels were selected. The shells were opened, and the soft tissues were removed. Sex was determined based on the color of the gonadal tissues. For each species–sex group, 24 individuals were randomly assigned to three non-overlapping pools, with eight individuals in each pool. The soft tissues from the eight individuals within each pool were combined to form one pooled biological replicate, resulting in three independent pooled biological replicates per group. Pooling was adopted to provide sufficient and homogeneous tissue for the multiple chemical analyses and to reduce the influence of individual-level heterogeneity, thereby obtaining a representative group-level flavor profile. Each pooled replicate was prepared and analyzed independently. The pooled samples were immediately frozen in liquid nitrogen and cryogenically homogenized into a fine frozen powder. The homogenized samples were stored at −80 °C prior to analysis. The samples were grouped as follows: male *M. coruscus* (MCM), female *M. coruscus* (MCF), male *M. galloprovincialis* (MGM), female *M. galloprovincialis* (MGF), male *P. viridis* (PVM), and female *P. viridis* (PVF).

High-performance liquid chromatography (HPLC)-grade solvents were purchased from Fisher Chemical (Fisher Scientific, Pittsburgh, PA, USA). Amino acid standards and the corresponding derivatization kit were obtained from Welch Materials Co., Ltd. (Shanghai, China). Nucleotide and organic acid standards were purchased from Tanmo Quality Inspection Technology Co., Ltd. (Beijing, China). Standard fatty acid methyl ester (FAME) mixtures were sourced from ANPEL Laboratory Technologies Inc. (Shanghai, China). Ultrapure water was prepared using a Milli-Q water purification system (Millipore, Bedford, MA, USA). All other chemicals and reagents used were of analytical grade and were purchased from Sinopharm Chemical Reagent Co., Ltd. (Shanghai, China).

### 2.2. Free Amino Acid Content Determination

FAA contents were analyzed following the methods employed by [26], using an HPLC system (1110, HITACHI, Tokyo, Japan) equipped with an Ultimate Amino Acid column (Welch Materials, 4.6 mm × 250 mm, 5 µm). For sample preparation, 2.5 g of the sample was weighed into a 25 mL volumetric flask and mixed with 10 mL of a 5% trichloroacetic acid solution. The mixture was sonicated for 10 min and allowed to precipitate for 2 h. After centrifugation at 10,000 rpm for 15 min, the supernatant was collected for further use. Derivatization was conducted using the Yuexu Amino Acid Derivatization Kit (Welch Materials Co., Ltd., Shanghai, China). Subsequently, 160 μL of the sample solution was added to a test tube together with 100 μL of each of derivatizing reagent A and derivatizing reagent B. The mixture was reacted at room temperature for 60 min. Subsequently, 400 μL of n-hexane was added; the tube was capped, shaken for 5–10 s and left to stand at room temperature until the layers separated. A 200 μL aliquot of the lower layer was transferred, diluted with 800 μL of water, and mixed thoroughly. Subsequently, 200 μL of the solution was transferred to a centrifuge tube, diluted with 800 μL of water, mixed thoroughly, and filtered through a 0.22 μm organic-phase filter membrane. The mobile phase consisted of mobile phase A and mobile phase B. Mobile phase A was 0.1 mol/L sodium acetate buffer (pH 6.50)–acetonitrile (93:7, *v*/*v*), and mobile phase B was acetonitrile–water (80:20, *v*/*v*). The chromatographic conditions were as follows: flow rate, 1.00 mL/min; detection wavelength, 254 nm; column temperature, 40 °C; and injection volume, 10 μL. FAAs were identified by comparing their retention times with those of amino acid standards and quantified using the external standard method on the basis of peak areas.

### 2.3. Taste Activity Value (TAV) Determination

The contribution of taste-active free amino acids to overall taste perception was evaluated using the taste activity value (TAV), the ratio of the concentration of each compound to its corresponding taste threshold [22].

(1) TAV=C/T

where C represents the concentration of each free amino acid in the sample, and T denotes the corresponding taste threshold reported in the literature. FAAs with TAV > 1 were considered to actively contribute to taste perception.

### 2.4. 5′-Nucleotide Content Determination

The 5′-nucleotide content was determined using an HPLC system (1260, Agilent Technologies, Santa Clara, CA, USA) equipped with a CAPCELL PAK C18 SG column (4.6 mm × 150 mm, Shiseido Co., Ltd., Tokyo, Japan) following a previously described method [27]. The mobile phase consisted of 20 mmol/L phosphoric acid, 20 mmol/L citric acid, and 40 mmol/L triethylamine. The flow rate was maintained at 1.00 mL/min, with detection performed at a wavelength of 260 nm. 5′-Nucleotides were identified by comparing their retention times with those of standards. Quantification was performed using external calibration curves constructed from nucleotide standards, and the results were calculated based on peak areas.

### 2.5. Organic Acid Content Determination

Extraction and analysis were conducted as previously described [27]. The organic acid content in the samples was determined using an HPLC system (1110, HITACHI, Tokyo, Japan) equipped with a C18 column (Atlantis^®^ T3, 3 μm, 4.6 mm × 150 mm, Waters Corporation, Milford, MA, USA). The mobile phase consisted of 0.02 mol/L disodium hydrogen phosphate, adjusted to pH 3 with phosphoric acid. The flow rate was set at 0.8 mL/min, the column temperature was maintained at 25 °C, and the detector wavelength was 210 nm. Organic acids were identified by comparing their retention times with those of standards. Quantification was performed using external calibration curves constructed from organic acid standards, and the results were calculated based on peak areas.

### 2.6. Equivalent Umami Concentration Determination

The equivalent umami concentration (EUC, g MSG/100 g) represents the amount of monosodium glutamate (MSG) required to produce a taste intensity equivalent to the combined effect of umami amino acids (UAAs) and nucleotides. This metric converts the umami taste intensity of a mixture containing umami amino acids (including aspartic acid (Asp) and glutamic acid (Glu)) and 5′-nucleotides (including inosine 5′-monophosphate (5′-IMP), guanosine 5′-monophosphate (5′-GMP), and adenosine 5′-monophosphate (5′-AMP)) into an equivalent concentration of MSG. The EUCs were calculated following an established methodology [28].

EUC (g MSG/100 g) = ∑a_i_b_i_ + 1218 (∑a_i_b_i_) (∑a_j_b_j_)

a_i_: UAA concentration (g/100 g) (Asp or Glu);

b_i_: UAA freshness coefficient relative to MSG (where Asp is 0.077 and Glu is 1.0);

a_j_: 5′-nucleotide concentration (g/100 g) (5′-IMP, 5′-GMP, 5′-AMP);

b_j_: taste nucleotide freshness coefficient relative to IMP (where 5′-IMP is 1.0, 5′-GMP is 2.3, and 5′-AMP is 0.18).

### 2.7. Fatty Acid Determination

Fatty acid analysis was conducted in accordance with Chinese standard GB 5009.168–2016 [29]. Briefly, the sample was subjected to acid hydrolysis with hydrochloric acid, and the released lipids were extracted using a petroleum ether–diethyl ether mixture. The extracted lipids were then saponified with sodium hydroxide–methanol solution under reflux in a water bath at 80 °C until the oil droplets disappeared. Boron trifluoride–methanol solution was then added, and the mixture was refluxed further in a water bath at 80 °C for 2 min to catalyze methylation and convert fatty acids into FAMEs. After methylation, FAMEs were extracted with n-heptane, and residual water in the organic phase was removed using anhydrous sodium sulfate.

The FAMEs were analyzed using a gas chromatograph equipped with a flame ionization detector (FID) (8890, Agilent Technologies, Santa Clara, CA, USA) and a DB-Fast FAME column (20 m × 0.18 mm i.d., 0.20 μm film thickness). The gradient temperature started at 80 °C, was increased to 220 °C at a rate of 25 °C/min (held for 3 min), and increased to 245 °C at a rate of 5 °C/min (held for 3 min). Nitrogen was used as the carrier gas, and the FID temperature was maintained at 250 °C. Fatty acids were identified by comparing their retention times with those of standard FAME mixtures (Anpel Laboratory Technologies (Shanghai) Inc., Shanghai, China). Relative fatty acid contents were quantified using the area normalization method and expressed as percentages of total fatty acids.

### 2.8. Gas Chromatography–Ion Mobility Spectrometry (GC–IMS) Analysis

VOCs in mussel tissue samples were identified using GC-IMS (FlavourSpec^®^, Gesellschaft für Analytische Sensorsysteme mbH, Dortmund, Germany) as previously described [28], with minor adjustments. A total of 2.00 g of mussel tissue sample was placed in a 20 mL headspace vial. The sample was incubated at 60 °C for 10 min with stirring at 500 rpm. Pure nitrogen was used as the carrier gas. The nitrogen flow program was as follows: 2 mL/min for 5 min, 100 mL/min for 5 min, and 150 mL/min for 7 min. For qualitative identification, the retention time (RT) and drift time (DT) of each compound were compared with reference data from the National Institute of Standards and Technology and ion mobility spectrometry (IMS) databases.

### 2.9. Statistical Analysis

All analyses were performed using three independent biological replicates, and the results are expressed as the mean ± standard deviation (SD) (n = 3). Each biological replicate consisted of pooled soft tissues from eight individual mussels, and the pooled biological replicate was used as the statistical unit. One-way analysis of variance followed by Duncan’s multiple range test was conducted using SPSS Statistics 26.0 (IBM Corp., Armonk, NY, USA). Graphs were generated using GraphPad Prism 10 (GraphPad Software, San Diego, CA, USA). Principal component analysis (PCA) and partial least squares–discriminant analysis (PLS-DA) were performed using MetaboAnalyst 6.0 (https://www.metaboanalyst.ca, accessed on 12 August 2026). Pearson correlation analysis among non-volatile flavor compounds was conducted using Origin Pro 2026 (OriginLab Corporation, Northampton, MA, USA) and visualized as correlation heatmaps. The heatmaps and correlation analyses between different classes of flavor-related compounds-including fatty acids, VOCs, amino acids, organic acids, and nucleotides-were generated using the ChiPlot online platform (https://www.chiplot.online/, accessed on 12 August 2026). Statistical significance was considered at *p* < 0.05.

## 3. Results and Discussion

### 3.1. Free Amino Acid Analysis

FAAs serve as essential precursors for the formation of aroma compounds, and the flavor characteristics of seafood are closely associated with FAA composition [30,31]. Categorized by their taste attributes, FAAs are typically classified into UAAs (Glu and Asp); sweet amino acids (SAAs) (glycine (Gly), alanine (Ala), threonine (Thr), proline (Pro), serine (Ser)); and bitter amino acids (BAAs) (histidine (His), arginine (Arg), methionine (Met), isoleucine (Ile), leucine (Leu), and lysine (Lys)) [22,32]. In the current study, 16 FAAs were identified and quantified across all mussel samples (Table S1), and representative chromatograms of the free amino acid standard mixture and a typical mussel sample are shown in Figure S2c,d.

**Figure 1:**
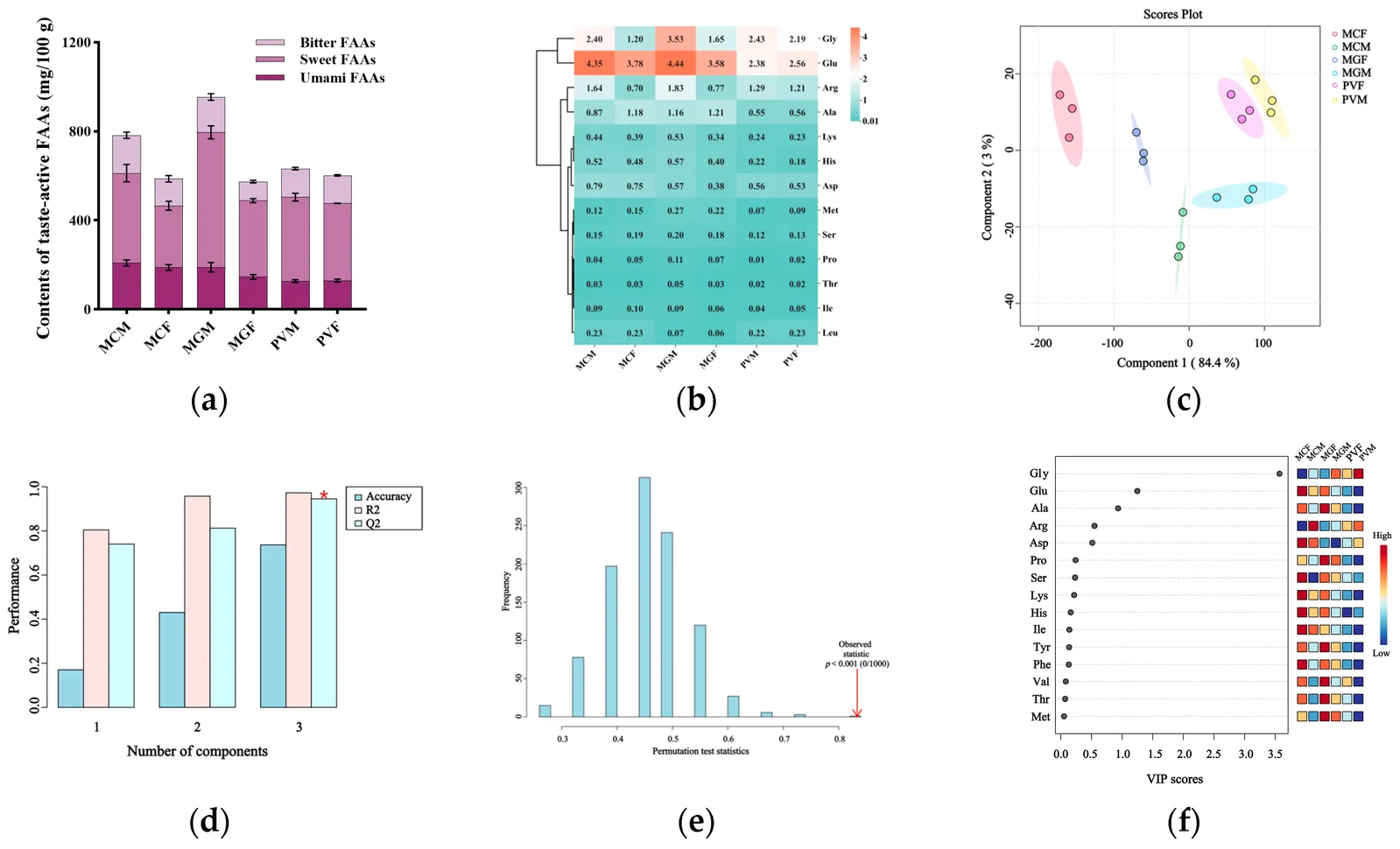
(**a**) Contents of taste-active free amino acids (FAAs); (**b**) taste activity value (TAV) heatmap; (**c**) partial least squares–discriminant analysis (PLS–DA) score plot; (**d**) Cross-validation (CV) results for model prediction accuracy. Q^2^ values (predictive ability) with asterisks (*) denote statistical significance (*p* < 0.05), indicating that the model outperforms random chance. (**e**) Permutation test statistics (n = 1000) validating model significance (*p* < 0.05); (**f**) variable importance in projection (VIP) scores plot highlighting key discriminant compounds.

In this study, SAAs were the predominant taste-active FAAs across all mussel samples, followed by UAAs and BAAs (Figure 1a), indicating that mussels generally possess a mild and pleasant taste profile. Among these species, *M. coruscus* exhibited the highest levels of UAAs (209.29 ± 13.31 mg/100 g, male and 188.36 ± 12.89 mg/100 g, female), and *M. galloprovincialis* contained the highest levels of SAAs (605.42 ± 28.72 mg/100 g, male and 342.67 ± 9.04 mg/100 g, female). Notably, SAAs content in *M. galloprovincialis* exceeded the combined levels of UAAs and BAAs, suggesting a greater contribution of sweet-tasting amino acids to its overall flavor profile. In addition, males consistently exhibited higher levels of UAAs, SAAs, and BAAs than females, consistent with a previous report [30]. In bivalves, gametogenesis and reproductive investment may alter amino acid metabolism and biochemical resource allocation, contributing to sex-related differences in FAA profiles [5,16].

The TAV heatmap revealed distinct differences in the profiles of taste-active amino acids among mussel species and sexes (Figure 1b). Glu and Gly showed TAV exceeding 1 in all samples, consistent with previous findings [33], indicating that they were the major taste-active amino acids in mussels. In addition, Arg and Ala exhibited TAV exceeding 1 in some samples, suggesting their potential contributions to the taste characteristics of specific groups. Notably, Arg exhibited TAV exceeding 1 in all male mussels, and a similar trend was reported by Li et al. [17]. Although generally regarded as a BAA, Arg can enhance flavor complexity and mouthfulness at moderate concentrations [34]. Consequently, the higher TAV of Arg in males may contribute to their enhanced taste intensity and fullness.

PLS-DA analysis was performed to evaluate differences in FAAs profiles among the six mussel groups. To evaluate the reliability of the PLS-DA model, fivefold cross-validation and permutation testing were performed. The optimal number of components was three based on the highest Q^2^ value, with accuracy = 0.736, R^2^ = 0.973, and Q^2^ = 0.951. Permutation testing (n = 1000) yielded *p* < 0.001, suggesting no apparent overfitting (Figure 1d,e). The first two latent variables in the model comprised 84.4% and 3.0% of the variance (totaling 87.4%) and clearly separated mussel groups in the score plot (Figure 1c).

The score plot showed that *P. viridis* was distinctly separated from the other two mussel species, whereas *M. coruscus* and *M. galloprovincialis* exhibited relatively similar FAAs profiles. Based on this model, variable importance in projection (VIP) scores (Figure 1f) were used to identify the major discriminative amino acids, including Glu, Gly, and Ala. A similar trend showing male mussels generally exhibited higher levels of several taste-active amino acids was reported by Chen et al. and Li et al. [16,17]. Compared with females, male mussels generally contained higher levels of Gly, Glu, Asp, and Arg; by contrast, female mussels generally contained higher levels of Ala than males (Table S1). Moreover, *M. coruscus* and *M. galloprovincialis* exhibited higher levels of UAAs than *P. viridis*, consistent with the EUC results. These variations are likely associated with species-specific metabolic characteristics and environmental factors, including food resources and habitat conditions [35].

### 3.2. 5′-Nucleotide Analysis

**Figure 2:**
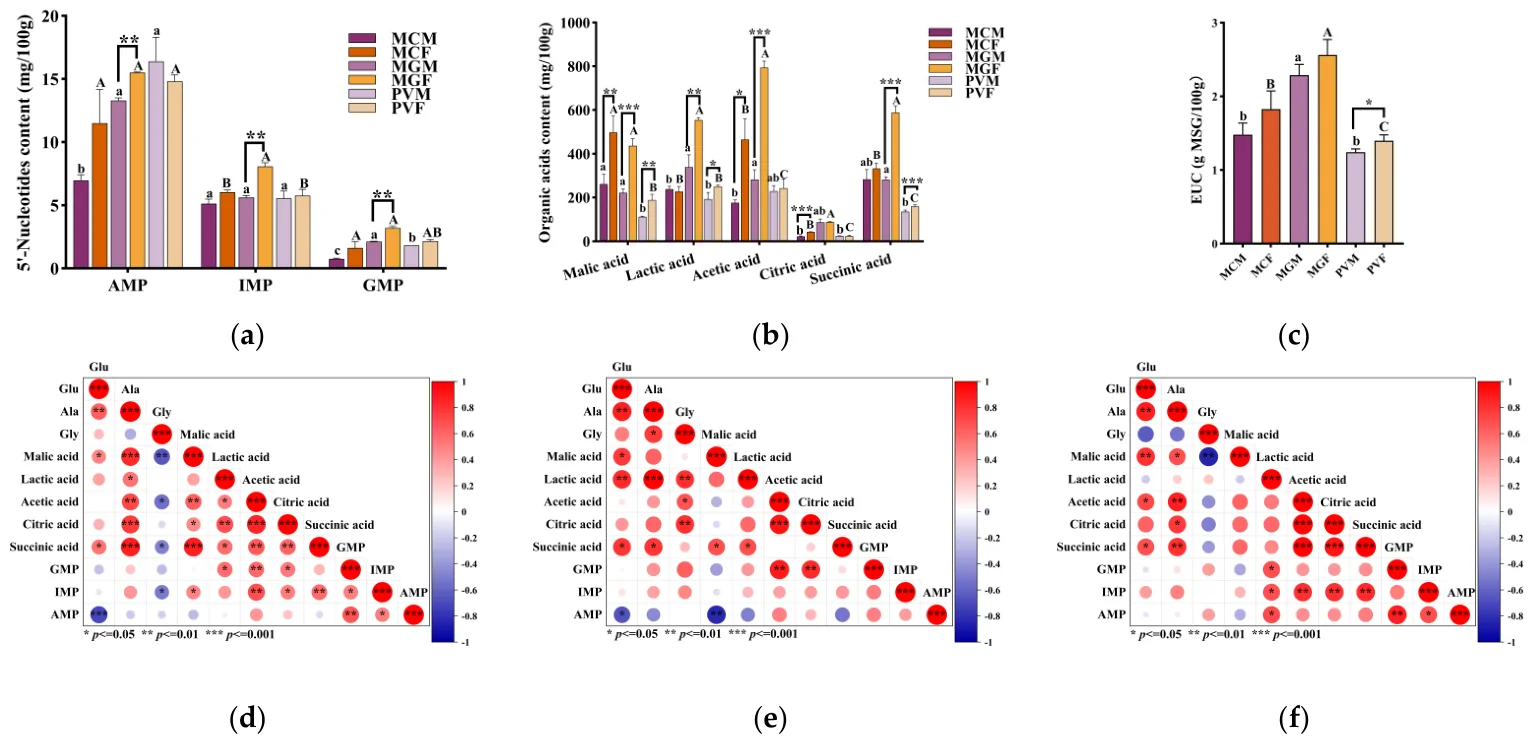
(**a**) 5′-nucleotides; (**b**) organic acids; (**c**) equivalent umami concentration (EUC); (**d**) Pearson correlation heatmap of non-volatile taste-active compounds among species; (**e**) Pearson correlation heatmap of non-volatile taste-active compounds in male mussels; (**f**) Pearson correlation heatmap of non-volatile taste-active compounds in female mussels. Note: In (**a**–**c**) asterisks (*) indicate differences between males and females within the same mussel species. * represents *p* < 0.05, ** represents *p* < 0.01, *** represents *p* < 0.001. Different lowercase superscript letters (a, b, c) denote significant differences among males (*p* < 0.05), whereas different uppercase superscript letters (A, B, C) indicate significant differences among females (*p* < 0.05).

5′-Nucleotides are important flavor-enhancing compounds that contribute to umami perception and synergistically interact with FAAs to intensify umami taste [33,36]. Three umami-related nucleotides—AMP, IMP, and GMP—were detected in all mussel samples (Figure 2a). AMP predominated among the detected nucleotides, followed by IMP and GMP, consistent with [30,32]. AMP is typically known as the umami-dominant nucleotide in oysters and squids, endowing aquatic products with good saltiness and sweetness and inhibiting bitterness [37]. IMP alone contributes modestly to taste but, with glutamate, markedly enhances umami intensity through synergistic interactions. AMP and IMP may also jointly improve freshness perception; while even low levels of IMP can increase taste complexity and impart pleasant savory and slightly sweet notes, GMP provides a meaty taste that is considerably stronger than that of other nucleotides [38].

Significant sex-related differences in nucleotide contents were observed only in *M. galloprovincialis*, with female mussels containing significantly higher levels of AMP, IMP, and GMP than male mussels (*p* < 0.05). The AMP, IMP, and GMP contents were 15.51 ± 0.05 mg/100 g, 8.08 ± 0.29 mg/100 g, 3.19 ± 0.13 mg/100 g in MGF and 13.29 ± 0.21 mg/100 g, 5.62 ± 0.16 mg/100 g, and 2.1 ± 0.04 mg/100 g in MGM. A similar pattern was also found, with females exhibiting higher levels than males [30]. By contrast, no significant sex-dependent differences were detected in *M. coruscus* or *P. viridis*. Notably, MGF exhibited the highest concentrations of all three nucleotides among the six mussel groups, indicating a pronounced accumulation of taste-active nucleotides in this sample. The higher 5′-nucleotide levels in female mussels confer a superior umami flavor. The elevated levels of free amino acids and taste-active nucleotides suggest that these compounds can potentially interact synergistically to enhance the overall flavor of mussels [13].

### 3.3. Organic Acid Analysis

Organic acids are important metabolites derived from glycogen metabolism in aquatic animals and play essential roles in flavor formation, quality maintenance, and nutritional regulation [37,39]. As shown in Figure 2b, five organic acids were detected in all mussel samples. Interspecific analysis revealed that MGM had higher levels of lactic acid (338.64 ± 56.53 mg/100 g), acetic acid (281.93 ± 44.79 mg/100 g), and citric acid (86.34 ± 14.89 mg/100 g), whereas MCM contained higher levels of malic acid (260.87 ± 45.68 mg/100 g), and succinic acid (282.86 ± 45.16 mg/100 g). Compared with males, females generally had higher levels of organic acids, particularly MGF, which exhibited the highest levels of lactic acid (553.39 ± 11.39 mg/100 g), acetic acid (793.79 ± 29.71 mg/100 g), citric acid (87.7 ± 1.65 mg/100 g), and succinic acid (587.56 ± 31.4 mg/100 g). The higher levels of these organic acids in female mussels may contribute to enhanced sour–umami synergistic effects and more complex flavor characteristics [17].

Intraspecific comparisons revealed significant sex differences in all four organic acids in *M. galloprovincialis*, except for citric acid. *M. coruscus* also showed significant sex differences in malic acid, acetic acid, and citric acid. Significant differences in malic acid, lactic acid, and succinic acid were observed within *P. viridis*. Lactic acid and succinic acid are key flavor-enhancing acids in glycolysis and together contribute to the unique flavor profile of seafood. Consistent with nucleotide-related patterns, the organic acid profiles further confirmed cultivar-specific differences. Previous studies in crustaceans and mollusks have demonstrated that lactic acid enhances flavor complexity through interactive effects with other taste-active compounds, particularly amino acids and nucleotides [19,40].

### 3.4. Equivalent Umami Concentration (EUC) Analysis

EUC represents the concentration of MSG required to produce an umami intensity equivalent to the synergistic interaction between UAAs and 5′-nucleotides [28,36]. As shown in Figure 2c, female mussels generally exhibited higher EUCs than male mussels, indicating a stronger synergistic umami effect. Among all samples, *M. galloprovincialis* showed the highest EUC values in both males and females, particularly in MGF, suggesting that this species possessed the strongest umami intensity. These results were consistent with the higher contents of UAAs and 5′-nucleotides observed in MG samples. Significant sex-dependent differences were only observed in *P. viridis*, with PVF exhibiting a significantly higher EUC than PVM. The elevated EUCs demonstrated that the synergistic interaction between Glu, Asp, and 5′-nucleotides significantly influenced the characteristic umami taste of mussels [34].

### 3.5. Correlation Analysis of Non-Volatile Components in Different Mussels

Pearson correlation analysis revealed both common and sex-dependent association patterns among non-volatile flavor compounds (Figure 2d–f). In all groups, organic acids exhibited strong positive correlations with each other, which may be associated with the umami and sweetness of these seafood products [41]. Similarly, IMP and GMP consistently exhibited positive correlations. IMP serves as the primary flavor precursor in raw food, whereas GMP enhances umami taste in food [42]. In addition, Ala and Glu maintained a positive correlation across all datasets, suggesting that their accumulation is regulated by similar metabolic pathways [19].

Despite these common features, the correlation networks varied markedly between the sexes. In males, Glu, Ala, and Gly formed a tightly connected amino acid module, and Gly positively correlated with lactic, acetic, and citric acid. These relationships suggest a close coupling between amino acid accumulation and energy metabolism [42]. Combined with the pronounced levels of UAAs, SAAs, and BAAs in males, the results emphasize the importance of protein turnover and amino acid metabolism in flavor formation [5].

By contrast, females exhibited a distinct correlation pattern. Gly negatively correlated with both Glu and Ala, whereas organic acids positively correlated with IMP and GMP. Citric, malic, and succinic acid are key intermediates of the TCA cycle, whereas IMP and GMP originate from ATP degradation. These correlations suggest a tighter linkage between energy metabolism and nucleotide transformation in females. This pattern may be associated with the higher energetic demands of ovarian development and nutrient storage [19,30].

Overall, sex-related differences were more pronounced than species-related variations. Male mussels were characterized by stronger amino acid and organic acid associations, whereas females exhibited closer interactions between organic acids and umami nucleotides. FAAs, organic acids and nucleotides contribute to flavor and serve as vital energy-yielding metabolites [42]. These findings suggest that males preferentially accumulate taste-active amino acids, whereas females rely more heavily on nucleotide metabolism, driving distinct mechanisms of non-volatile flavor formation.

### 3.6. Fatty Acid Analysis

Lipids function as essential precursors in the development of food aroma and flavor, while also modulating the sensory properties of other taste-active compounds. Polyunsaturated fatty acids (PUFAs), particularly EPA and DHA, are closely associated with seafood flavor because they act as critical precursors of volatile aroma compounds [43,44,45]. Representative chromatograms of the fatty acid standard mixture and a typical mussel sample are shown in Figure S2a,b.

**Figure 3:**
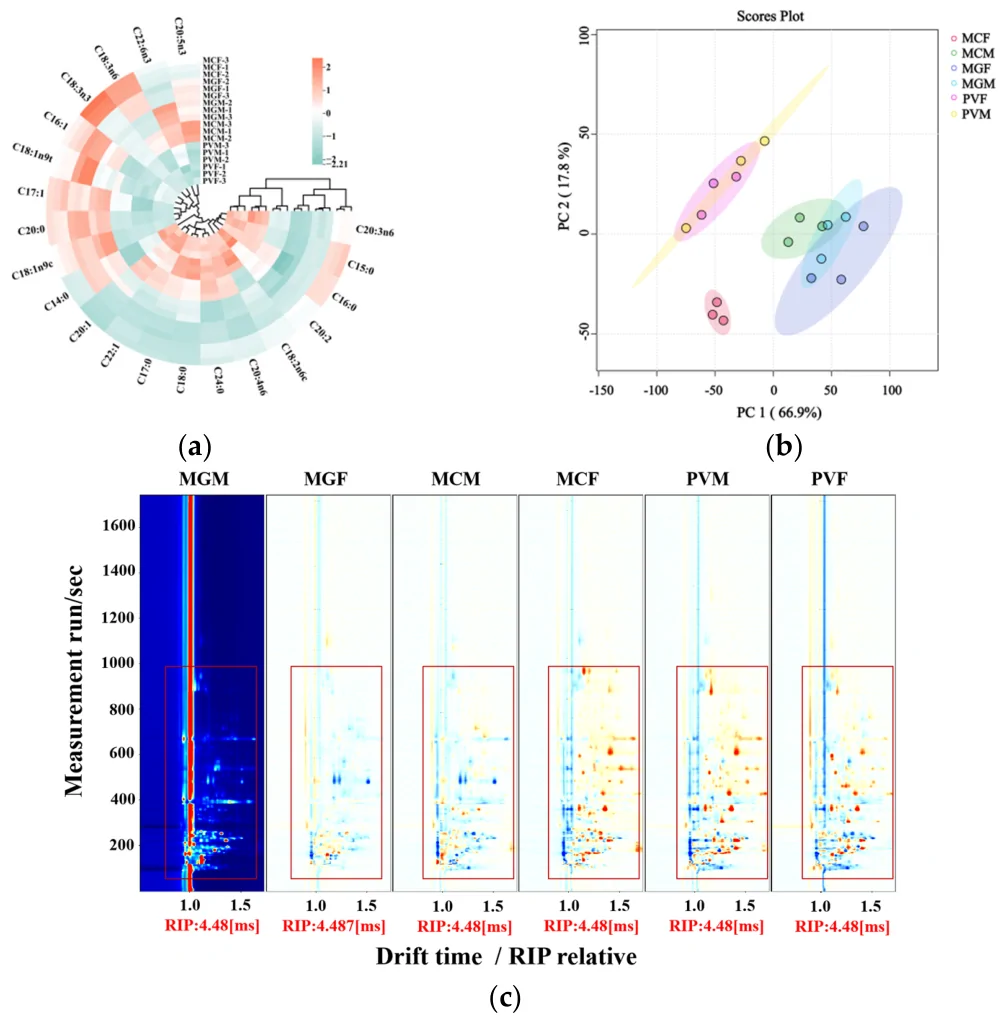
(**a**) Heatmap of FAMEs; (**b**) principal component analysis (PCA) based on gas chromatography–ion mobility spectrometry (GC–IMS) volatile profiles; (**c**) Vertical view of the GC-IMS two-dimensional topographic plot. The red boxes in panel (**c**) indicate the main regions where volatile organic compound (VOC) signals were distributed and compared among the different mussel groups.

As shown in Table S2, PUFAs (42.45–51.87%) were significantly more abundant than monounsaturated fatty acids (MUFAs, 12.12–21.71%) and saturated fatty acids (SFAs, 32.75–41.88%). ω-3 PUFAs predominated across all mussel samples, with DHA and EPA comprising more than 90% of total ω-3 PUFAs, consistent with previous reports on marine bivalves [6,46,47]. Significant differences in fatty acid compositions were observed among mussel species and sexes (Figure 3a). MCM exhibited the highest proportions of total polyunsaturated fatty acids (ΣPUFA) (51.87 ± 0.21%), EPA (22.28 ± 0.33%), and ω-3 PUFAs (43.13 ± 0.25%), and MGM showed the highest DHA (20.67 ± 0.46%) proportion. By contrast, PVM contained relatively elevated levels of ω-6 PUFAs (10.62 ± 0.28%). Significant differences in DHA proportions occurred among the three mussel species, whereas significant differences in EPA proportions were confined mainly within *M. galloprovincialis*. PUFAs are readily degraded by lipid oxidation, yielding hydroperoxides that subsequently decompose into a myriad of secondary oxidation products, including hydrocarbons, vinyl alcohols, oxyesters, alkenals, alkadienals, and vinyl ketones, among others [43,48].

C16:0 has been reported in numerous studies as the primary SFAs in mussels, followed by stearic acid (C18:0) [12]. The primary components of MUFAs include palmitoleic acid (C16:1), oleic acid (C18:1n9), and EPA (C20:1n9). Significant sex-related and interspecific differences were observed among the three mussel species, with C16:1 being the most abundant MUFA. In this study, only the C16:1 content of the MGF (12.85%) was consistent with the levels reported by Fuentes et al. [49]; the content in the other species was below this range. Fatty acid composition is influenced by environmental conditions, available phytoplankton resources, and nutrient composition [8,33]. *P. viridis* was collected from the southern coast of China, where higher ambient temperatures and more abundant zooplankton resources may contribute to its higher SFAs content. Biandolino et al. [50] used C16:1 and EPA content as indicators of diatom consumption. In the current study, the proportion of C16:1 in *M. galloprovincialis* was higher than in *M. coruscus* and *P. viridis*, but sex differences varied among species. Intraspecific analysis revealed that females had a higher C16:1 proportion than males, suggesting that female mussels rely more on diatoms as a food source, potentially linked to their reproductive strategy. Therefore, the characteristic flavor variations can potentially be attributed to differences in fatty acid compositions among mussel species and sexes.

### 3.7. Volatile Compounds in Different Mussels

#### 3.7.1. Odor Profile Analysis of Different Mussels

The PCA diagram of the volatile components in various mussels is presented in Figure 3b. PC1 and PC2 constituted 66.7% and 17.9% of the total variance, respectively. PVM and PVF clustered closely, as did MCM, MGM, and MGF. By contrast, MCF separated most distinctly from the other mussel groups, indicating a distinct olfactory profile. Variations in the VOCs of various mussels may be directly compared using the topographic plot of GC–IMS fingerprinting. As seen in Figure 3c, differences between the treated groups were assessed with the MGM as the reference, subtracting other mussels from it in accordance with the original methodology. After applying the “Verify” function, the initial background color shifted to white; red indicated a higher VOC concentration than the MGM reference, whereas blue represented a lower concentration.

Compared with the reference group MGM, MGF and MCM showed lower relative VOC signal intensities, as indicated by the dark blue regions in Figure 3c, whereas MCF, PVM, and PVF exhibited higher relative abundances of several VOCs, as reflected by the red signals. MCF showed the highest MUFA content, whereas PVM and PVF had relatively higher SFA contents, suggesting that differences in lipid composition contribute to the distinct volatile flavor profiles among mussel groups [33,42]. In oysters, aldehydes, ketones, and alcohols are major volatile groups, and their profiles vary with cultivar, geographic origin, and harvest season [15].

#### 3.7.2. Qualitative Analysis of Odor Profiles in Different Mussels

Variations in VOCs across various species and sexes were further investigated based on their standard retention indices. Color intensity reflected the amount of VOCs, with deeper hues indicating higher concentrations. As shown in Table S3, 67 VOCs were found, including monomers (M) and dimers (D). These VOCs comprised 17 aldehydes, 12 alcohols, 9 ketones, 3 organic acids, 6 ethers, 2 furans, 2 sulfur, 3 nitrogen, and 13 unidentified compounds.

**Figure 4:**
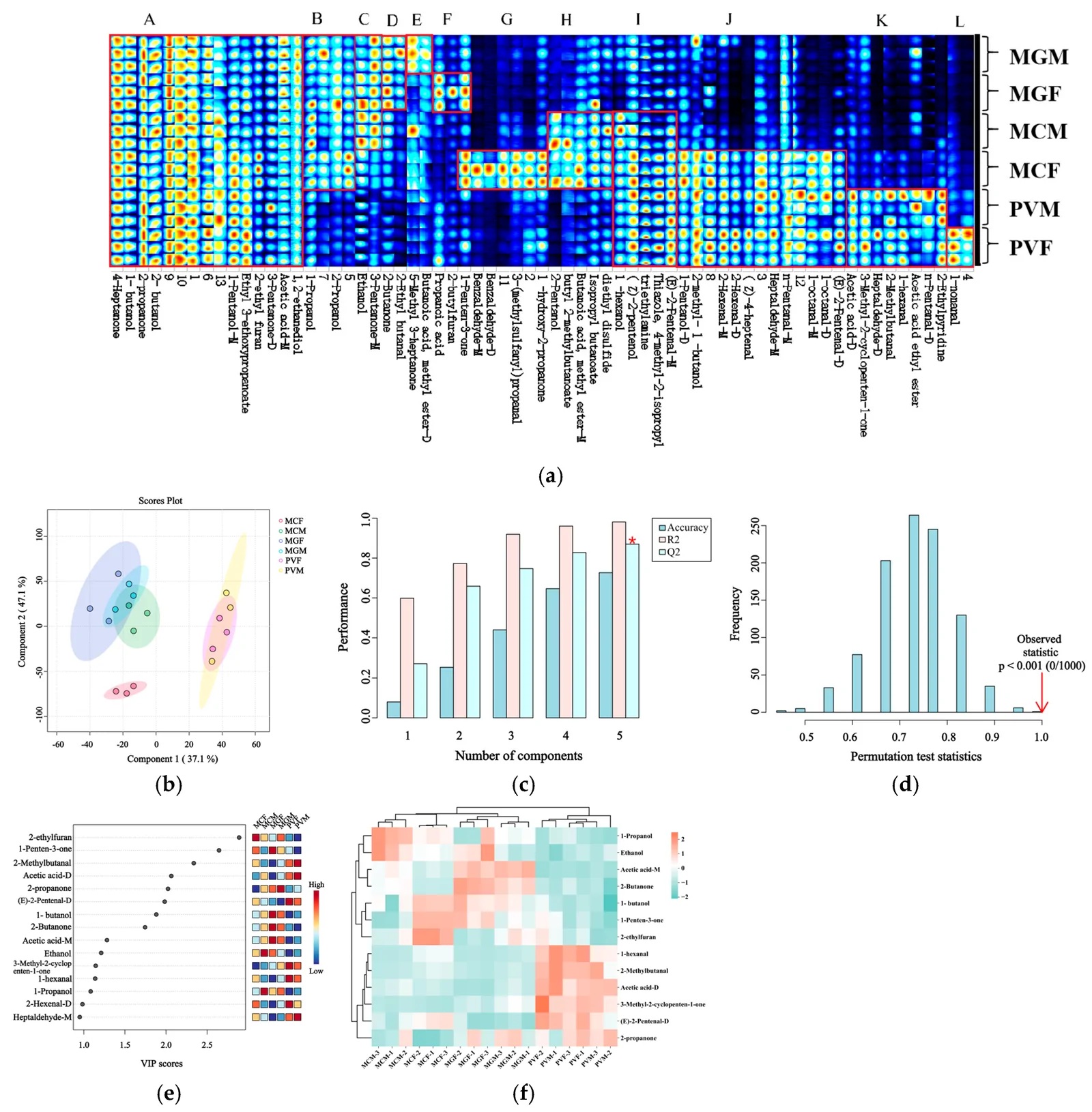
(**a**) Gallery plot generated from GC–IMS data. Letters A–L indicate selected volatile organic compound (VOC) regions with distinct signal distribution patterns among the six mussel groups. The compounds corresponding to each region are shown along the x-axis. (**b**) partial least squares–discriminant analysis (PLS–DA) score plot of VOC profiles; (**c**) Cross-validation (CV) results indicate model prediction accuracy. Q^2^ values (predictive ability) with asterisks (*) denote statistical significance (*p* < 0.05), indicating that the model outperforms random chance. (**d**) Permutation test statistics (n = 1000) validating model significance (*p* < 0.05) (**e**) VIP scores plot highlighting key discriminant compounds; (**f**) Hierarchical clustering heatmap of differential volatile compounds (VIP > 1).

All mussel samples varied in species and sampling locations but shared a similar volatile profile characterized by fruity, fatty, and grassy aroma–related compounds (Figure 4a, region A), reflecting the common biochemical characteristics of marine bivalves [4,49]. Mussel samples exhibited distinct species- and sex-dependent differences in volatile profiles. MGM was characterized by abundant esters and ketones, resulting in pronounced fruity and sweet aroma notes (Figure 4a, regions D and E). MGF shared similar fruity attributes but contained higher levels of propanoic acid and 1-penten-3-one, which introduced pungent and fermented nuances (Figure 4a, region F). MCM presented a balanced aroma profile rich in fruity, fatty, and green notes (Figure 4a, regions H and I), whereas MCF expressed fermented, sulfurous, and amine-like odors associated with sulfur-containing compounds and amino acid degradation products (Figure 4a, regions I and J). PVM and PVF were characterized by higher abundances of aldehydes and alcohols (Figure 4a, regions I–K). Specifically, PVF contained elevated levels of nonanal, hexanal, and octanal, driving its intense fatty, waxy, citrus-like, and floral aroma profile. Overall, *M. galloprovincialis* was characterized by higher relative abundances of VOCs associated with sweet–fruity and ester-like odor notes, *M. coruscus* showed relatively higher levels of VOCs associated with fermented and sulfurous notes, whereas *P. viridis* exhibited higher relative abundances of aldehydes and related VOCs associated with green and fatty odor notes. Female samples generally contained higher levels of aldehydes and alcohols than males, resulting in more complex and intense aroma profiles. Aldehydes, ketones, esters, and alcohols constituted the major volatile compounds detected in the samples. Owing to their low odor thresholds, aldehydes primarily determine seafood aroma, whereas alcohols are linked to characteristic fatty and meaty notes [23,51]. Most aldehydes, ketones, and alcohols originate from lipid oxidation and amino acid metabolism, contributing to the fruity, fatty, and green aroma characteristics [32]. As intermediates of Maillard reactions, furans may further enhance aroma complexity through the formation of heterocyclic compounds [27]. The remaining ethers, amines, and other compounds were detected in smaller quantities and exerted no dominant effect on the flavor profile. The contribution of odorants is determined by their types and thresholds [19].

#### 3.7.3. PLS–DA Analysis of Odor Profiles in Different Mussels

PLS–DA was performed based on the signal intensities of the identified volatile compounds (Figure 4b). The first two latent variables in the model comprised 37.1% and 47.1% of the variance (totaling 84.2%). Similar to the PCA results, the samples were clearly separated by species and sex, indicating distinct differences in volatile flavor profiles. To evaluate the reliability of the PLS–DA model, fivefold cross-validation and permutation testing were performed. The optimal number of components was three based on the highest Q^2^ value, with accuracy = 0.727, R^2^ = 0.981, and Q^2^ = 0.870. Permutation testing (n = 1000) yielded *p* < 0.001, suggesting no apparent overfitting (Figure 4c,d).

The corresponding VIPs were subsequently used to identify the key volatile compounds contributing to sample discrimination [52]. As shown in Figure 4e, Thirteen key volatile signals, corresponding to 12 compounds, were identified, including 3 aldehydes (2-methylbutanal, (E)-2-pentenal, and hexanal), 4 ketones (1-penten-3-one, 2-propanone, 2-butanone, and 3-methyl-2-cyclopenten-1-one), 3 alcohols (ethanol, 1-propanol, and 1-butanol), 2-ethylfuran, and acetic acid, which was detected as both monomer and dimer signals. Hierarchical clustering analysis based on these differential VOCs identified 2 distinct groups (Figure 4f). The *P. viridis* samples were characterized by higher levels of hexanal, 2-methylbutanal, acetic acid-D, 3-methyl-2-cyclopentene-1-one, (E)-2-pentenal-D, and 2-propanone, whereas the remaining samples were enriched in 1-propanol, ethanol, 2-butanone, acetic acid-M, 1-butanol, and 1-penten-3-one. Among all groups, MCF exhibited the highest levels of 1-butanol, 1-penten-3-one, and 2-ethylfuran, which may be associated with alcohol-like, pungent/fishy, and smoky/burnt odor notes, respectively [53].

These differential compounds were primarily associated with lipid oxidation, amino acid degradation, and fermentation-related pathways [19,33]. Hexanal, (E)-2-pentenal, and 1-penten-3-one serve as typical oxidation products of PUFAs and impart grassy, fatty, and marine-like aromas [54]. Similarly, 2-ethylfuran may be generated through the oxidation of ω-3 PUFAs and has been associated with caramel-like or butterscotch aroma characteristics. 2-Methylbutanal, derived from amino acid degradation, imparts malty and nutty notes; ethanol, 1-propanol, and 1-butanol elicit fermented and fruity characteristics. Acetic acid yields acidic and pungent notes, and 3-methyl-2-cyclopentene-1-one is linked to caramel-like and roasted aromas. The combined effects of these compounds drive the distinct aroma characteristics observed across mussel species and sexes. The enrichment of aldehydes in *P. viridis* suggests a greater contribution of lipid oxidation-derived aromas [55], whereas the higher abundance of alcohols and ketones in *M. coruscus* and *M. galloprovincialis* may lead to more pronounced fermented, fruity, and fatty flavor characteristics.

#### 3.7.4. Interactions Among Fatty Acids, Volatile Compounds and Non-Volatile Flavor Substances in Different Mussels

**Figure 5:**
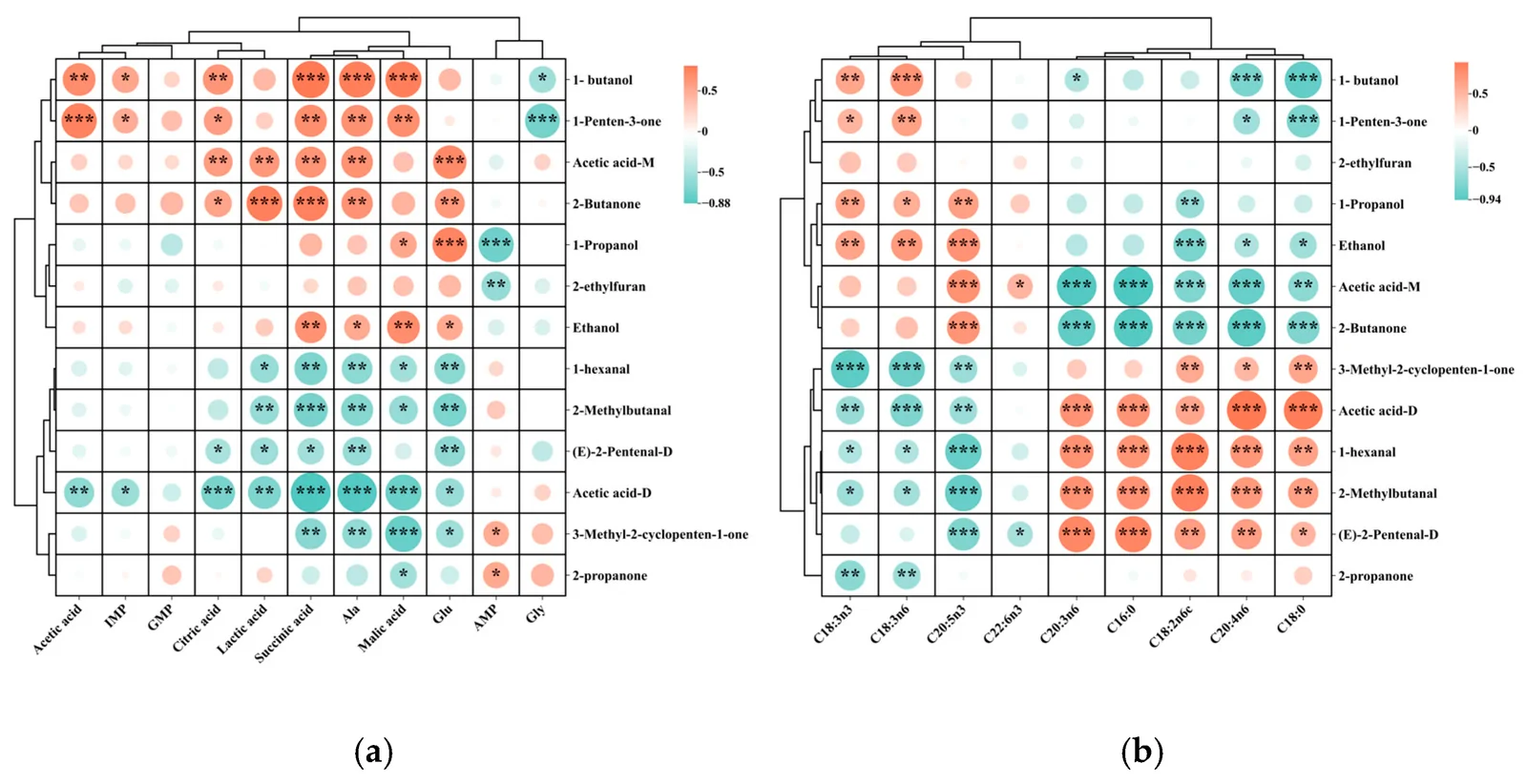
(**a**) Pearson correlation heatmap between non-volatile flavor compounds (free amino acids, organic acids, and nucleotides) and differential volatile compounds; (**b**) Pearson correlation heatmap between unsaturated fatty acids and differential volatile compounds. Asterisks indicate the significance levels of Pearson correlation coefficients: *, *p* < 0.05; **, *p* < 0.01; ***, *p* < 0.001.

Correlation analysis revealed extensive interactions among fatty acids, volatile compounds, and non-volatile flavor substances. Moreover, the interactions between volatile and non-volatile flavor compounds segregated into two distinct VOC clusters (Figure 5a), confirming the close association between aroma formation and taste-related metabolism [56]. The alcohol, ketone rich cluster, including 1-butanol, 1-penten-3-one, 2-butanone, 1-propanol, and ethanol, negatively correlated with AMP and Gly but was positively associated with most other non-volatile compounds. This pattern indicates an inverse association between AMP- and Gly-related taste accumulation and the relative abundance of these volatile signals. Conversely, the second cluster, represented by hexanal, 2-methylbutanal, (E)-2-pentenal-D, acetic acid-D, 3-methyl-2-cyclopentene-1-one, and 2-propanone, exhibited broad positive correlations with the majority of non-volatile metabolites. These relationships suggest potential links between VOC profiles and amino acid transformation, nucleotide degradation, and organic acid-related energy metabolism [57]. Similar associations among volatile compounds, lipid oxidation, nucleotide degradation, and amino acid metabolism have been reported in aquatic products [58,59]. The contrasting behaviors of AMP and Gly further suggest that these two compounds do not accumulate synchronously with other taste-active metabolites during flavor development. Overall, these findings indicate that mussel flavor profiles are associated with coordinated changes in volatile aroma compounds and non-volatile taste metabolites rather than isolated changes in individual flavor components [17].

Correlation analysis between volatile and fatty acid compounds delineated two major VOC clusters (Figure 5b). Differential volatile compounds formed two distinct clusters. The first comprised mainly alcohols and ketones, showing positive correlations with most non-volatile flavor compounds and a strong association with ω-3 PUFAs, including EPA and DHA. This pattern was consistent with the higher proportions of ω-3 PUFAs and the greater relative abundances of alcohols and ketones in *M. coruscus* and *M. galloprovincialis*, which may contribute to their fermented, fruity, and marine-like aroma notes. By contrast, the second cluster, dominated by aldehydes such as hexanal and (E)-2-pentenal, positively correlated with ω-6 PUFAs, particularly C18:2n-6 and C20:4n-6. This correlation was consistent with the fatty acid composition and volatile profile of *P. viridis*, which contained higher levels of ω-6 PUFAs and accumulated more aldehydes, potentially contributing to its grassy and fatty aroma characteristics. These results suggest that lipid oxidation can potentially be a key pathway linking fatty acid composition with the formation of aldehydes, alcohols, ketones, and other volatile compounds in mussels. Moreover, different classes of fatty acids may be associated with distinct volatile profiles through different oxidation-related pathways [45,48,60].

Overall, these findings suggest that flavor formation in mussels is not determined by a single class of compounds but is associated with coordinated variations in fatty acids, volatile compounds, and non-volatile taste-active substances. Notably, species–associated differences in ω-3 and ω-6 PUFAs may contribute to, rather than directly drive, the formation of characteristic aroma profiles in mussels [61].

## 4. Conclusions

This study comprehensively evaluated the volatile and non-volatile flavor profiles of three commercially important mussel species, *M. coruscus*, *M. galloprovincialis*, and *P. viridis*, with consideration of sex-related differences. Significant species- and sex-dependent differences were observed in free amino acids, organic acids, 5′-nucleotides, fatty acids, and volatile compounds. SAAs predominated across all samples, followed by UAAs, and Glu, Gly, and Ala contributed substantially to taste perception. *M. coruscus* and *M. galloprovincialis* showed higher umami potential than *P. viridis*, as indicated by their higher EUC values.

GC–IMS analysis revealed distinct volatile profiles among mussel groups. Among the three species, *M. galloprovincialis* exhibited a VOC profile enriched in compounds previously associated with sweet, fruity, and ester-like odor notes, whereas *M. coruscus* showed relatively higher levels of compounds associated with fermented and sulfurous notes. *P. viridis* was mainly characterized by higher relative abundances of aldehydes and related VOCs, which may be linked to green and fatty odor notes. Key differential volatile compounds mainly included aldehydes, ketones, alcohols, 2-ethylfuran, and acetic acid. Correlation analysis indicated potential associations among fatty acids, volatile compounds, and non-volatile taste metabolites. In particular, ω-3 PUFAs were more closely associated with alcohols and ketones, whereas ω-6 PUFAs were more closely associated with aldehydes. These findings indicate that mussel flavor profiles may be associated with coordinated variations in fatty acid composition, amino acid metabolism, nucleotide metabolism, organic acid-related energy metabolism, and volatile compound profiles.

Several limitations should be acknowledged. First, because the three mussel species were collected from different geographical locations, species and geographic origin were not independently controlled. Therefore, the observed differences should be interpreted as combined effects of species, sex, and culture environment rather than species or sex alone. Second, although GC–IMS is rapid and suitable for volatile fingerprinting, compound identification mainly relies on retention index and drift time matching, and signal intensity is more appropriate for relative comparison than absolute quantification. Thus, further GC–MS-based targeted analysis is needed to confirm the identities and concentrations of key VOCs. Third, sensory evaluation was not performed in this study; therefore, the sensory relevance of the identified volatile and non-volatile compounds requires further validation by trained sensory evaluation or consumer testing.

## Data Availability

The original contributions presented in this study are included in the article/supplementary material. Further inquiries can be directed to the corresponding authors.

## Supplementary Materials

The following supporting information can be downloaded at https://www.mdpi.com/article/10.3390/foods15162915/s1: Figure S1: Representative gross images and hematoxylin and eosin staining gonadal tissue sections of male and female *M. coruscus*; Figure S2: Representative GC-FID and HPLC chromatograms of analytical standards and mussel samples. (a) 37-component FAME standard mixture; Table S1: Differences in free amino acids between males and females of three mussel species; Table S2: Differences in fatty acid composition content between males and females of three mussel species (% of total fatty acids); Table S3: Differences in volatile flavor compounds between males and females of three mussel species.
