# Supplementary material for: Characterization of Species- and Sex-Dependent Volatile and Non-Volatile Flavor Profiles in Three Commercially Important Mussel Species
Source: Foods. 2026 Aug 20;15(16):2915. doi: 10.3390/foods15162915 (PMC13511765; doi:10.3390/foods15162915)
Supplement: Supplementary file 1 [file foods-15-02915-s001.zip › foods-4424816-supplementary.pdf]

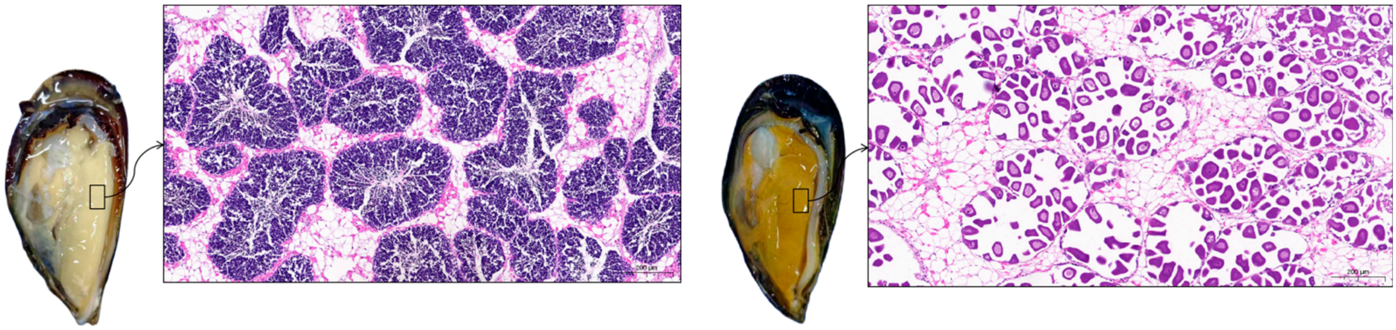

(a) (b)

Figure S1. Representative gross images and hematoxylin and eosin staining gonadal tissue sections of male and female *M. coruscus*. (a) Male *M. coruscus*; (b) female *M. coruscus*. The boxed areas indicate the corresponding sampling regions for histological observation. Scale bars = 200 μm.

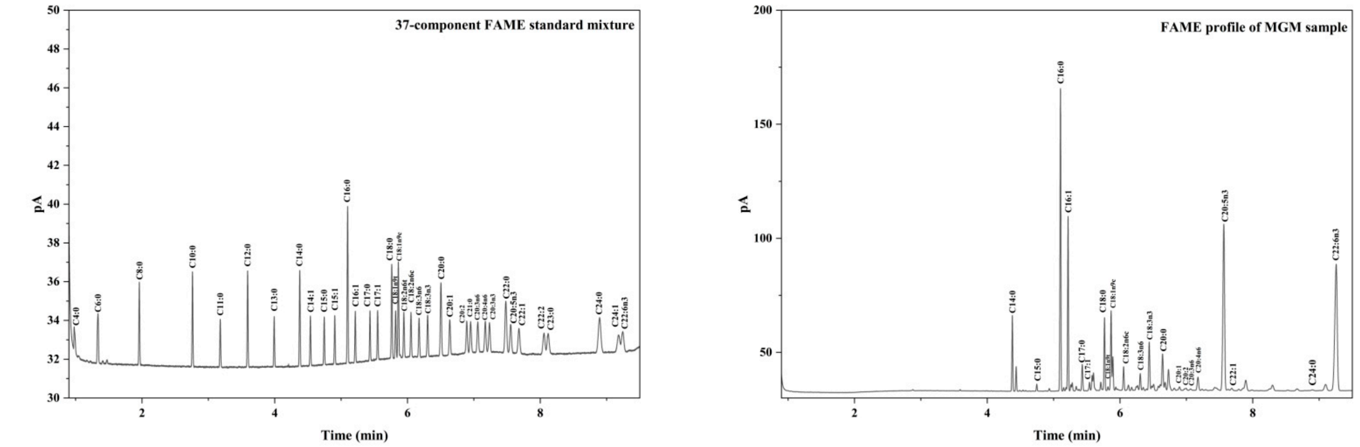

(a) (b)

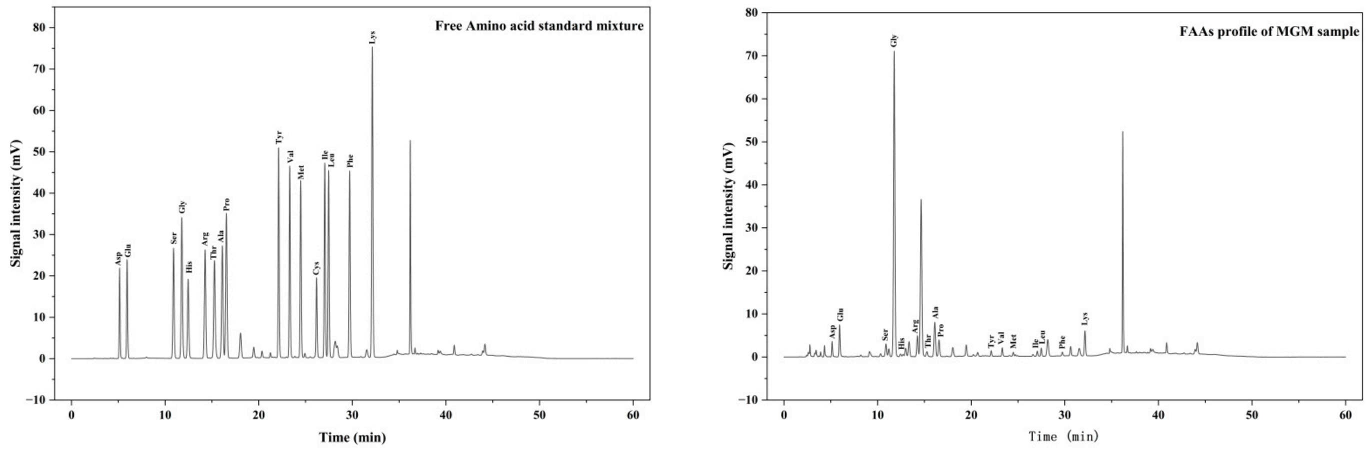

(c) (d)

Figure S2. Representative GC-FID and HPLC chromatograms of analytical standards and mussel samples. (a) 37-component FAME standard mixture; (b) FAME profile of the MGM sample; (c) FAAs standard mixture; (d) FAAs profile of the MGM sample. Note: MGM, male *Mytilus galloprovincialis*.

Table S1

Differences in free amino acid between males and females of three mussel species

FAAs	FAAs (mg/100 g)									Taste	TAVs					
	MCM	MCF	*p*-value	MGM	MGF	*p*-value	PVM	PVF	*p*-value	threshold	MCM	MCF	MGM	MGF	PVM	PVF
Asp	78.89 ± 5.86^{a}	74.81 ± 1.43^{A}	0.351	56.66 ± 10.53^{b}	38.49 ± 5.85^{C}	0.059	56.38 ± 4.82^{b}	53.19 ± 4.18^{B}	0.435	100	0.79	0.75	0.57	0.38	0.56	0.53
Glu	130.40 ± 10.78^{a}	113.54 ± 12.14^{A}	0.146	133.26 ± 10.5^{a}	107.51 ± 5.99^{A}	0.021	71.26 ± 1.33^{b}	76.66 ± 2.18^{B}	0.021	30	4.35	3.78	4.44	3.58	2.38	2.56
Ser	21.80 ± 2.13^{b}	28.64 ± 3.56^{A}	0.046	30.68 ± 5.66^{a}	26.79 ± 0.90^{A}	0.305	17.79 ± 1.40^{b}	18.83 ± 0.80^{B}	0.326	150	0.15	0.19	0.20	0.18	0.12	0.13
Thr	6.71 ± 0.85^{a}	7.89 ± 1.36^{A}	0.271	12.29 ± 2.68^{a}	8.96 ± 0.61^{A}	0.158	4.53 ± 0.14^{a}	5.47 ± 0.42^{B}	0.021	260	<0.1	<0.1	<0.1	<0.1	<0.1	<0.1
Ala	52.13 ± 9.85^{a}	70.66 ± 7.44^{A}	0.060	69.76 ± 6.34^{a}	72.37 ± 1.22^{A}	0.551	33.27 ± 0.77^{b}	33.69 ± 3.86^{B}	0.868	60	0.87	1.18	1.16	1.21	0.55	0.56
Pro	10.80 ± 0.90^{b}	14.42 ± 0.96^{B}	0.009	33.34 ± 4.42^{a}	20.51 ± 0.83^{A}	0.008	4.41 ± 0.28^{c}	4.59 ± 0.22^{C}	0.425	300	<0.1	<0.1	0.11	<0.1	<0.1	<0.1
Gly	311.98 ± 25.83^{b}	155.38 ± 7.17^{C}	0.001	459.36 ± 20.38^{a}	214.04 ± 8.26^{B}	<0.0001	316.53 ± 17.33^{b}	284.08 ± 3.86^{A}	0.034	130	2.40	1.20	3.53	1.65	2.43	2.19
His	10.44 ± 2.09^{a}	9.62 ± 0.89^{A}	0.567	11.37 ± 1.62^{a}	7.92 ± 1.17^{A}	0.041	4.37 ± 0.26^{b}	3.51 ± 0.59^{B}	0.080	20	0.52	0.48	0.57	0.40	0.22	0.18
Arg	81.94 ± 9.60^{a}	34.89 ± 2.67^{B}	0.001	91.52 ± 2.60^{a}	38.48 ± 4.93^{B}	<0.0001	64.64 ± 6.04^{b}	60.3 ± 2.62^{A}	0.318	50	1.64	0.70	1.83	0.77	1.29	1.21
Met	3.46 ± 0.62^{b}	4.39 ± 0.62^{B}	0.139	8.12 ± 1.22^{a}	6.70 ± 0.67^{A}	0.152	2.13 ± 0.13^{b}	2.66 ± 0.43^{C}	0.113	30	0.12	0.15	0.27	0.22	0.07	0.09
Ile	8.38 ± 0.70^{a}	9.25 ± 1.06^{A}	0.306	8.19 ± 1.51^{a}	5.56 ± 0.47^{B}	0.045	3.72 ± 0.37^{b}	4.07 ± 0.62^{B}	0.456	90	0.09	0.10	0.09	0.06	0.04	0.05
Leu	43.54 ± 8.71^{a}	44.25 ± 9.08^{A}	0.926	13.63 ± 2.37^{b}	10.45 ± 1.22^{B}	0.108	42.09 ± 4.19^{a}	44.03 ± 4.36^{A}	0.609	190	0.23	0.23	0.07	0.06	0.22	0.23
Lys	21.86 ± 1.79^{a}	19.27 ± 1.56^{A}	0.132	26.61 ± 5.93^{ab}	16.90 ± 2.06^{A}	0.055	11.81 ± 0.51^{b}	11.59 ± 0.25^{B}	0.524	50	0.44	0.39	0.53	0.34	0.24	0.23
Val	9.06 ± 0.94^{a}	9.36 ± 0.82^{A}	0.696	13.03 ± 1.60^{a}	9.55 ± 0.46^{A}	0.022	3.98 ± 0.09^{b}	5.04 ± 0.72^{B}	0.122							
Phe	5.73 ± 1.36^{b}	7.88 ± 1.25^{AB}	0.115	9.66 ± 0.56^{a}	11.91 ± 3.22^{A}	0.299	4.00 ± 0.35^{b}	6.31 ± 2.08^{B}	0.130							
Tyr	7.36 ± 1.14^{b}	10.46 ± 1.24^{A}	0.033	12.43 ± 1.08^{a}	9.28 ± 0.72^{A}	0.014	4.48 ± 0.15^{c}	5.29 ± 0.38^{B}	0.027							
UAA	209.29 ± 13.31^{a}	188.36 ± 12.89^{A}	0.122	189.92 ± 20.95^{a}	146.00 ± 10.29^{B}	0.031	127.65 ± 6.11^{b}	129.86 ± 6.36^{B}	0.687							
SAA	403.42 ± 38.82^{b}	276.99 ± 19.76^{B}	0.007	605.42 ± 28.72^{a}	342.67 ± 9.04^{A}	<0.0001	376.54 ± 17.31^{b}	346.68 ± 1.32^{AB}	0.095							
BAA	169.61 ± 14.06^{a}	121.67 ± 14.84^{A}	0.015	159.44 ± 14.87^{a}	86.01 ± 6.26^{B}	0.001	128.78 ± 5.78^{b}	126.15 ± 2.72^{A}	0.516							
TFAA	1269.55 ± 71.99^{a}	1249.98 ± 47.44^{A}	0.714	1362.75 ± 73.78^{a}	1236.02 ± 18.25^{A}	0.045	1057.28 ± 26.8^{b}	1042.46 ± 8.86^{B}	0.415							

Note: Concentration data are presented as mean ± SD. *p*-values indicate the differences between males and females of the same mussel species. Different superscript lowercase letters (a, b, c) denote significant differences among males ($p < 0.05$), while different superscript uppercase letters (A, B, C) denote significant differences among females ($p < 0.05$). The full names and abbreviations of amino acids are as follows. Aspartic acid (Asp), Glutamine (Glu), Serine (Ser), Glycine (Gly), Histidine(His), Arginine (Arg), Threonine (Thr), Alanine (Ala), Proline (Pro), Valine (Val), Methionine (Met), Isoleucine (Ile), Leucine (Leu), Lysine (Lys), Tyrosine (Tyr), Phenylalanine (Phe), Taurine (Tau), TFAA= total free amino acids. Umami amino acids (UAA): Asp+Glu ;Sweet amino acids (SAA): Ser+Thr+Ala+Pro+Gly; Bitter amino acids (BAA): His+Arg+Met+Ile+Leu+Lys.

Table S2

Differences in fatty acid composition content between males and females of three mussel species (% of total fatty acids)

compositions (%)	MCM	MCF	*p*-value	MGM	MGF	*p*-value	PVM	PVF	*p*-value
C14:0	1.51 ± 0.02^{c}	2.88 ± 0.12^{B}	<0.0001	4.20 ± 0.23^{a}	4.70 ± 0.12^{A}	0.028	3.26 ± 0.16^{b}	5.42 ± 0.36^{A}	0.001
C15:0	0.56 ± 0.01^{b}	0.78 ± 0.03^{B}	<0.0001	0.44 ± 0.01^{c}	0.56 ± 0.01^{C}	<0.0001	0.78 ± 0.02^{a}	0.86 ± 0.02^{A}	0.008
C16:0	24.58 ± 0.18^{b}	25.32 ± 0.06^{A}	0.002	18.62 ± 0.94^{c}	21.86 ± 0.22^{B}	0.004	25.73 ± 0.16^{a}	25.54 ± 0.27^{A}	0.342
C17:0	2.57 ± 0.03^{b}	1.76 ± 0.04^{B}	<0.0001	1.97 ± 0.12^{c}	1.66 ± 0.06^{B}	0.015	3.10 ± 0.03^{a}	2.75 ± 0.08^{A}	0.003
C18:0	6.34 ± 0.11^{b}	3.27 ± 0.08^{B}	<0.0001	4.95 ± 0.21^{c}	2.91 ± 0.04^{C}	<0.0001	9.79 ± 0.38^{a}	7.42 ± 0.48^{A}	0.003
C20:0	1.41 ± 0.06^{b}	3.00 ± 0.17^{B}	<0.0001	3.54 ± 0.29^{c}	4.12 ± 0.11^{A}	0.031	1.00 ± 0.07^{a}	1.26 ± 0.05^{C}	0.006
C24:0	0.42 ± 0.03^{b}	0.23 ± 0.02^{B}	0.001	0.11 ± 0.00^{c}	0.16 ± 0.01^{C}	0.004	0.42 ± 0.03^{a}	0.38 ± 0.03^{A}	0.202
∑SFA	37.38 ± 0.22^{b}	37.23 ± 0.11^{B}	0.351	33.82 ± 0.50^{c}	35.98 ± 0.19^{C}	0.002	44.08 ± 0.07^{a}	43.63 ± 0.41^{A}	0.194
C16:1	4.38 ± 0.20^{b}	10.15 ± 0.82^{B}	<0.0001	9.68 ± 0.78^{a}	13.37 ± 0.36^{A}	0.002	5.16 ± 0.3^{b}	7.31 ± 0.41^{C}	0.002
C17:1	0.39 ± 0.01^{b}	0.51 ± 0.03^{A}	0.003	0.56 ± 0.03^{a}	0.46 ± 0.02^{B}	0.006	0.17 ± 0.01^{c}	0.27 ± 0.01^{C}	<0.0001
C18:1n9t	0.17 ± 0.04^{ab}	0.21 ± 0.01^{B}	0.145	0.19 ± 0.01^{a}	0.30 ± 0.02^{A}	0.001	0.14 ± 0.01^{b}	0.18 ± 0.01^{C}	0.014
C18:1n9c	1.46 ± 0.14^{a}	3.31 ± 0.24^{B}	<0.0001	5.07 ± 0.40^{b}	4.05 ± 0.15^{A}	0.014	1.61 ± 0.06^{a}	1.66 ± 0.19^{C}	0.687
C20:1	3.96 ± 0.11^{a}	2.74 ± 0.04^{B}	<0.0001	3.31 ± 0.22^{b}	2.89 ± 0.06^{B}	0.073	3.59 ± 0.17^{b}	4.07 ± 0.19^{A}	0.031
C22:1	0.49 ± 0.02^{a}	0.21 ± 0.01^{B}	<0.0001	0.23 ± 0.02^{c}	0.17 ± 0.01^{C}	0.01	0.44 ± 0.02^{b}	0.63 ± 0.01^{A}	<0.0001
∑MUFA	10.83 ± 0.41^{b}	17.12 ± 1.14^{B}	0.001	19.02 ± 0.98^{a}	21.24 ± 0.47^{A}	0.024	11.12 ± 0.46^{b}	14.13 ± 0.72^{C}	0.004
C18:2n6c	1.37 ± 0.05^{c}	1.90 ± 0.01^{B}	<0.0001	1.68 ± 0.12^{b}	1.52 ± 0.06^{C}	0.092	2.95 ± 0.22^{a}	2.55 ± 0.12^{A}	0.049
C18:3n6	0.76 ± 0.03^{a}	1.10 ± 0.01^{A}	<0.0001	0.48 ± 0.02^{b}	0.85 ± 0.01^{B}	<0.0001	0.35 ± 0.02^{c}	0.47 ± 0.03^{C}	0.002
C18:3n3	1.33 ± 0.04^{a}	2.76 ± 0.12^{A}	<0.0001	1.26 ± 0.07^{ab}	1.32 ± 0.02^{B}	0.287	1.15 ± 0.06^{b}	1.04 ± 0.03^{C}	0.034
C20:2	0.59 ± 0.02^{a}	0.57 ± 0.01^{B}	0.184	0.34 ± 0.03^{b}	0.44 ± 0.02^{C}	0.003	0.60 ± 0.01^{a}	0.88 ± 0.05^{A}	0.001
C20:3n6	0.22 ± 0.01^{b}	0.26 ± 0.01^{B}	0.001	0.15 ± 0.01^{c}	0.19 ± 0.01^{C}	0.006	0.34 ± 0.03^{a}	0.31 ± 0.03^{A}	0.218
C20:4n6	3.72 ± 0.07^{b}	2.25 ± 0.04^{B}	<0.0001	1.42 ± 0.11^{c}	1.12 ± 0.02^{C}	0.04	4.49 ± 0.07^{a}	4.03 ± 0.04^{A}	0.001
C20:5n3 EPA	23.31 ± 0.32^{a}	19.08 ± 0.57^{B}	<0.0001	20.37 ± 0.33^{b}	20.36 ± 0.26^{A}	0.959	16.68 ± 0.43^{c}	17.13 ± 0.72^{C}	0.405
C22:6n3 DHA	20.47 ± 0.15^{b}	17.72 ± 0.38^{A}	<0.0001	21.45 ± 0.48^{a}	16.97 ± 0.33^{A}	<0.0001	18.24 ± 0.30^{c}	15.83 ± 0.63^{B}	0.004
∑PUFA	51.18 ± 0.24^{a}	45.07 ± 1.05^{A}	0.001	46.82 ± 0.57^{b}	42.34 ± 0.63^{B}	0.001	44.21 ± 0.41^{c}	41.36 ± 0.80^{B}	0.005
ω3	45.12 ± 0.22^{a}	39.57 ± 1.07^{A}	0.001	43.08 ± 0.57^{b}	38.65 ± 0.58^{A}	0.001	36.08 ± 0.66^{c}	34.01 ± 0.88^{B}	0.031
ω6	6.07 ± 0.06^{b}	5.50 ± 0.01^{B}	0.000	3.74 ± 0.03^{c}	3.69 ± 0.07^{C}	0.312	8.13 ± 0.30^{a}	7.36 ± 0.12^{A}	0.015

Note: Concentration data are presented as mean ± SD. *p*-values indicate the differences between males and females of the same mussel species. Different superscript lowercase letters (a, b, c) denote significant differences among males ($p < 0.05$), while different superscript uppercase letters (A, B, C) denote significant differences among females ($p < 0.05$).

Table S3

Differences in volatile flavor compounds between males and females of three mussel species

Count	Category	Compound	CAS#	Formula	MW	RI	Rt [sec]	Dt [a.u.]
1	Alcohols	1– butanol	71–36–3	C4H10O	74.1	1156.2	391.534	1.38238
2		1–Propanol	71–23–8	C3H8O	60.1	1041.7	264.258	1.11302
3		Ethanol	64–17–5	C2H6O	46.1	938.1	192.429	1.13301
4		2–Propanol	67–63–0	C3H8O	60.1	940.5	193.843	1.22299
5		2–butanol	78–92–2	C4H10O	74.1	1038.6	261.713	1.15301
6		2–methyl–1–butanol	137–32–6	C5H12O	88.1	1218.2	480.486	1.23321
7		1 –hexanol	111–27–3	C6H14O	102.2	1367.1	715.295	1.32953
8		1–Pentanol–M	71–41–0	C5H12O	88.1	1254.8	537.483	1.25212
9		1–Pentanol–D	71–41–0	C5H12O	88.1	1253.9	535.966	1.51162
10		2–Pentanol	6032–29–7	C5H12O	88.1	1117.4	339.717	1.20639
11		(Z)–2–pentenol	1576–95–0	C5H10O	86.1	1337.4	668.651	1.44712
12		1,2–ethanediol	107–21–1	C2H6O2	62.1	1647.6	1319.863	1.09557
13	Aldehydes	1–nonanal	124–19–6	C9H18O	142.2	1394.8	761.347	1.4804
14		1–octanal–M	124–13–0	C8H16O	128.2	1295.8	608.414	1.41278
15		1–octanal–D	124–13–0	C8H16O	128.2	1295.5	607.989	1.81796
16		Heptaldehyde–M	111–71–7	C7H14O	114.2	1180.1	427.425	1.33621
17		Heptaldehyde–D	111–71–7	C7H14O	114.2	1179.8	426.85	1.68988
18		n–Pentanal–M	110–62–3	C5H10O	86.1	985.4	222.032	1.18204
19		n–Pentanal–D	110–62–3	C5H10O	86.1	985.6	222.155	1.4239
20		1–hexanal	66–25–1	C6H12O	100.2	1086.3	303.368	1.27157
21		(E)–2–Pentenal–M	1576–87–0	C5H8O	84.1	1132.9	359.553	1.10939
22		(E)–2–Pentenal–D	1576–87–0	C5H8O	84.1	1131.4	357.634	1.35777
23		(Z)–4–heptenal	6728–31–0	C7H12O	112.2	1243.4	519.141	1.14569
24		2–Hexenal–M	505–57–7	C6H10O	98.1	1218.1	480.367	1.17807
25		2–Hexenal–D	505–57–7	C6H10O	98.1	1216.2	477.654	1.51402
26		2–Methylbutanal	96–17–3	C5H10O	86.1	925.2	185.101	1.40276
27		2–Ethyl butanal	97–96–1	C6H12O	100.2	997.7	230.568	1.50672
28		Benzaldehyde–M	100–52–7	C7H6O	106.1	1510.5	967.125	1.15498
29		Benzaldehyde–D	100–52–7	C7H6O	106.1	1510.7	967.571	1.47188
30	Ketones	1–Penten–3–one	1629–58–9	C5H8O	84.1	1026.1	251.815	1.31171
31		2–Butanone	78–93–3	C4H8O	72.1	912.3	178.007	1.24548
32		2–propanone	67–64–1	C3H6O	58.1	843	144.354	1.11802
33		3–Pent anone–M	96–22–0	C5H10O	86.1	984.3	221.274	1.11177
34		3–Pentanone–D	96–22–0	C5H10O	86.1	984.7	221.557	1.3567
35		5–Methyl 3–heptanone	541–85–5	C8H16O	128.2	1275.1	571.919	1.28274
36		4–Heptanone	123–19–3	C7H14O	114.2	1157.1	392.813	1.58154

Count	Category	Compound	CAS#	Formula	MW	RI	Rt [sec]	Dt [a.u.]
37		3–Methyl–2–cyclopenten–1–one	2758–18–1	C6H8O	96.1	1499.8	943.903	1.11045
38		1–hydroxy–2–propanone	116–09–6	C3H6O2	74.1	1312.4	631.706	1.22673
39	Esters	Butanoic acid, methyl ester–M	623–42–7	C5H10O2	102.1	1009.2	238.927	1.1472
40		Butanoic acid, methyl ester–D	623–42–7	C5H10O2	102.1	994.8	228.551	1.43347
41		Acetic acid ethyl ester	141–78–6	C4H8O2	88.1	895.5	169.18	1.33595
42		Isopropyl butanoate	638–11–9	C7H14O2	130.2	1036.8	260.299	1.25673
43		butyl 2–methylbutanoate	15706–73–7	C9H18O2	158.2	1256.6	540.531	1.38287
44		Ethyl 3–ethoxypropanoate	763–69–9	C7H14O3	146.2	1337.8	669.291	1.22163
45	Organic acids	Acetic acid–M	64–19–7	C2H4O2	60.1	1471.5	889.926	1.05697
46		Acetic acid–D	64–19–7	C2H4O2	60.1	1469.4	886.142	1.16136
47		Propanoic acid	29102	C3H6O2	74.1	1565.2	1094.7	1.10715
48	Furans	2–ethyl furan	3208–16–0	C6H8O	96.1	957.4	204.023	1.04554
49		2–butylfuran	4466–24–4	C8H12O	124.2	1141.4	370.989	1.18303
50	Sulfur	3–(methylsulfanyl)propanal	3268–49–3	C4H8OS	104.2	1466.4	880.73	1.40074
51		diethyl disulfide	110–81–6	C4H10S2	122.2	1217.2	479.097	1.13101
52	Nitrogen	2–Ethylpyridine	100–71–0	C7H9N	107.2	1287.7	594.47	1.11058
53		Thiazole, 4–methyl–2–isopropyl	15679–13–7	C7H11NS	141.2	1337.8	669.208	1.63027
54		triethylamine	121–44–8	C6H15N	101.2	781.5	119.873	1.0869
55	Others	1	unidentified	*	0	1254.6	537.129	1.28664
56		2	unidentified	*	0	1219.6	482.623	1.40564
57		3	unidentified	*	0	1254.3	536.672	1.61177
58		4	unidentified	*	0	1279.2	579.106	1.11531
59		5	unidentified	*	0	1115	336.71	1.08919
60		6	unidentified	*	0	1023.6	249.836	1.14176
61		7	unidentified	*	0	941.9	194.691	1.37294
62		8	unidentified	*	0	887.8	165.281	1.20299
63		9	unidentified	*	0	795.5	125.074	1.11962
64		10	unidentified	*	0	1012.9	241.75	1.02674

Count	Category	Compound	CAS#	Formula	MW	RI	Rt [sec]	Dt [a.u.]
65		11	unidentified	*	0	997.7	230.568	1.31489
66		12	unidentified	*	0	984.4	221.369	1.38914
67		13	unidentified	*	0	905.1	174.148	1.0266

Note：M, monomer; D, dimer; T, trimer; numbers indicate unidentified compounds.
